# Creating a Corpus of Multilingual Parent-Child Speech Remotely: Lessons Learned in a Large-Scale Onscreen Picturebook Sharing Task

**DOI:** 10.3389/fpsyg.2021.734936

**Published:** 2021-11-19

**Authors:** Fei Ting Woon, Eshwaaree C. Yogarrajah, Seraphina Fong, Nur Sakinah Mohd Salleh, Shamala Sundaray, Suzy J. Styles

**Affiliations:** ^1^Psychology, Nanyang Technological University, Singapore, Singapore; ^2^Centre for Research and Development in Learning, Nanyang Technological University, Singapore, Singapore; ^3^Agency for Science, Technology and Research, Singapore Institute for Clinical Sciences, Singapore, Singapore

**Keywords:** online assessment, multilingualism, corpus creation, parent-child interaction, book sharing

## Abstract

With lockdowns and social distancing measures in place, research teams looking to collect naturalistic parent-child speech interactions have to develop alternatives to in-lab recordings and observational studies with long-stretch recordings. We designed a novel micro-longitudinal study, the Talk Together Study, which allowed us to create a rich corpus of parent-child speech interactions in a fully online environment (N participants = 142, N recordings = 410). In this paper, we discuss the methods we used, and the lessons learned during adapting and running the study. These lessons learned cover nine domains of research design, monitoring and feedback: Recruitment strategies, Surveys and Questionnaires, Video-call scheduling, Speech elicitation tools, Videocall protocols, Participant remuneration strategies, Project monitoring, Participant retention, and Data Quality, and may be used as a primer for teams planning to conduct remote studies in the future.

## Introduction

Singapore is a diverse environment for studying language acquisition, with 74.3% of the population reporting literacy in two or more languages (Department of Statistics of Singapore, 2021). In late 2019, our team was preparing for a large-scale project to create a corpus of 500 linguistically diverse home-recordings, and document patterns of language switching, mixing and translanguaging over the first 4 years of life. In the original research plan, a visit to the family home would initiate a series of audio recordings, including high fidelity recording of the parent’s voice in their different languages, a parent-child interaction centred on a picture-book narration task, and a day-long ambient speech recording using a baby-worn recording device [e.g., a Language ENvironment Analysis (LENA) device (Gilkerson and Richards, 2008)].

As cases of COVID-19 emerged in Singapore in January 2020, parents began declining invitations to participate in face-to-face procedures. Strict lockdown measures from 8 April, 2020 precluded visits to family homes (Ministry of Health Singapore, 2020). With our corpus-building goals in mind, we designed a novel study that would allow us to develop a large corpus of parent-child speech. The Talk Together Study is a remote micro-longitudinal study in which parent-child dyads participated in an online book-sharing session at three time-points, separated by at least 1 month. Embedded in the micro-longitudinal design was a Randomised Control Trial (RCT) of an intervention to enhance child-directed talk through “daily tips”. We preregistered the RCT and a target of 150 participants (for further details, see Sundaray et al., 2021). The adapted design allowed us to create a large corpus of parent-child interactions (typically around 20 min in duration) using a novel, remote procedure. This report describes the strategies adopted and lessons learned while running the study.

In recent years, interest in online behavioural studies has been increasing, with conferences and workshops dedicated to online methods (e.g., BeOnline Conference, 2021) and the emergence of user-friendly interfaces for building online experiments (e.g., Gorilla Online Experiment Builder: Anwyl-Irvine et al., 2019) and managing participant recruitment (e.g., Amazon, 2005^1^; Prolific, 2014^2^). Commonly noted advantages of online research include the possibility of recruiting large samples in a short amount of time, as well as reducing physical barriers to participation for a diverse range of participants, and greater access for under-represented populations such as children, and speakers of non-WEIRD languages (Woods et al., 2015; Evershed, 2021; Nation, 2021). The emergence of the COVID-19 has accelerated transitions to online methods for many research groups.

In infancy research, one innovation has been the development of asynchronous browser-based methods that allow a parent to initiate an online study session in their own time, without synchronous involvement from a researcher. Examples include eye-gaze studies using e-Babylab (Lo et al., 2021) and looking-time studies using Lookit (Scott and Schulz, 2017), a platform that has even been used with infants as young as 7 months-of-age (Bochynska and Dillon, 2021). One innovative global collaboration – Manybabies-AtHome – aims to coordinate researchers around the world to develop and run online asynchronous tasks such as preferential looking paradigm and looking-while-listening in home-based tests of infants from diverse backgrounds and nationalities (Zaadnoordijk et al., 2021).

Compared to the efficiency of recruiting and running asynchronous online studies, synchronous online studies requiring a researcher to interact with the participants may have certain limitations, especially regarding manpower. However, synchronous online studies in which a researcher initiates the parent-child interaction, but does not participate, more closely simulate the role of a researcher in a lab-based study, who is able to check equipment function before beginning, and monitoring the study from a concealed location (e.g., behind a two-way mirror, over a live video feed).

In the study reported here, one of the primary goals was to create an audio and transcription corpus of parent-child interactions. While some asynchronous platforms allow participants to record and upload audio recordings asynchronously (e.g., Discoveries Online: Rhodes et al., 2000), the issue of data storage and transfer remains challenging for researchers working outside of the jurisdictions that govern these platforms (Scott and Schulz, 2017; Zaadnoordijk et al., 2021), and existing online research platforms do not provide high-fidelity in-application audio recording. In addition, in our own team’s pilot tests, the audio quality of unsupervised participant recordings varied greatly due to (1) variable background noise, and (2) variable recording devices and audio drivers, which may compress and degrade audio signals in various ways.

Given the primary goal of the current study was to build up a corpus of parent-child interactions, we developed new synchronous researcher-led online protocols to meet the need for data quality checks at the onset of recording. In this paper, we discuss methods we developed to create a corpus of parent-child speech in a fully online environment using a wordless picture book “Little Orangutan: What a Scary Storm” (Styles, 2020b). We provide concrete recommendations for teams who wish to conduct researcher-led synchronous online studies by video call.

## Methods

### Research Setting

The study was conducted fully online. In line with local health restrictions, at the start of the study, the research team were working from home and parents were recruited to participate from their own homes. Internet usage rates in Singapore are extremely high (International Telecommunication Union (ITU) World Telecommunication/ICT Indicators Database, 2020), with over 96% of young and middle-aged Singaporeans using the internet, more mobile data subscriptions than residents, and homes are connected by high bandwidth internet (Infocomm Media Development Authority, 2019a,b,c).

### Research Design

There were three time-points for data collection in our study – T1 (baseline), T2 (post-intervention), and T3 (post-intervention switch). At each time-point, parents completed a series of online surveys we had designed about their child’s language exposure, understanding, and use (Styles et al., 2021; Woon et al., 2021). After completion of all surveys at each time-point, the parent-child dyad participated in a recorded video call with a member of the Data Collection team.

### Eligibility Criteria

Posts on social media invited Singaporean parents with a child between the ages of 8 and 36 months to join the study. Instances where families took some time to enrol in the study post-consent, families with children up to 40 months of age were considered eligible to participate. Non-Singaporean parents who expressed interest were contacted by email and text messages to assess their eligibility. Since the primary targets of the corpus were local patterns of language use, families were deemed eligible if at least one parent had spent a significant time living in Singapore, and had completed most of their education in Singapore.

### Recruitment Strategies

We preregistered the study with a target sample size of 150 parent-child dyads and used rolling enrolment to replace any participants who dropped out before a preregistered recruitment stop date. Recruitment used organic reach on social media: Our primary strategy was posting announcements about the study on our lab’s Facebook page, with lab members sharing to their personal networks; our secondary strategy was to share these posts on local Facebook parenting groups. These peer-to-peer “mummy groups” are popular in Singapore as they allow parents to post questions, share their parenting woes, and seek advice from fellow parents. Each local Facebook group has around 5000 members, and each time we posted to one of these groups we saw increases in recruitment.

### Consent and Survey Chain

Parents who were interested in taking part in the study were directed to an online consent form in the survey platform, Qualtrics (2005) (Qualtrics, Provo, UT, United States). As informed consent includes an opportunity to ask questions (American Psychological Association, 2017), the online consent page included our lab’s contact details. Parents who provided personal details and contact information on the online consent form but did not complete the procedure were contacted by our team, and we answered any questions they had. We also monitored the lab’s social media posts and accounts. In all advertising materials, we shared a lab mobile phone number, used to answer queries during the consent process, and throughout the length of the study. The majority of inquiries we received were through asynchronous text-based messaging platforms (WhatsApp, Facebook messenger, or SMS text messages).

**FIGURE 1:**
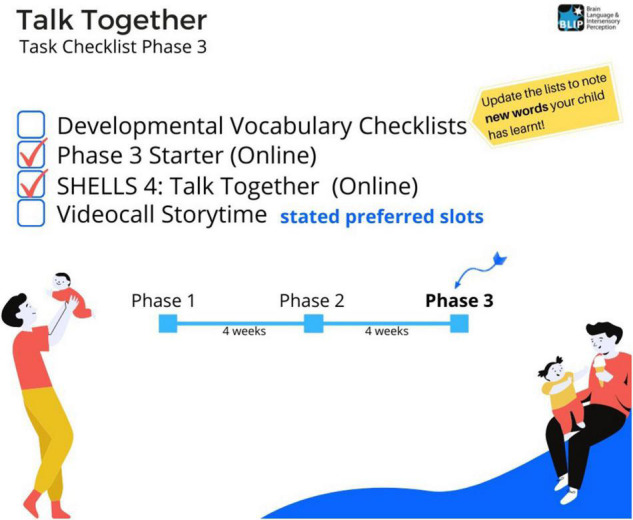
Example of a visual progress checklist to help parents track their progress through tasks at a particular time-point.

The consent form generated a unique random ID for each parent-child dyad. Parents were sent a series of surveys, linked through the identifier, making up a “chain”. Each survey was designed to be short (i.e., 5–20 min duration), so that parents could fit them in around other activities. On completion of each survey, a unique hyperlink was generated to the next survey in the chain. The hyperlink was displayed onscreen, along with a visual checklist of progress through the tasks for each time-point (see Figure 1). Survey completion also triggered reminder emails to the parent, and to the Data Collection team for monitoring.

Part of the study involved information about specific vocabulary items across the diverse languages of Singapore. We created an in-house vocabulary checklist for including questions about which words a child “understands” and “understands and also says” (c.f., MacArthur Bates CDI: Fenson et al., 1994). After piloting various formats for the checklist, the parent-preferred format was a PDF with clickable checkboxes, which was returned to the parent at subsequent time-points for updating (i.e., adding new words). Parents in our study were asked to return the completed PDFs by email replies.

### Video Call Scheduling and Pre-call Checks

The online survey chain ended with a form for parents to select a video call appointment. Parents were sent a text message and were phoned by the Data Collection team to confirm the timeslot. Parents who had not completed the preliminary tasks were followed up with reminder phone calls and text messages. Parents who did not complete the required tasks within 8 weeks were removed from the main study.

### Speech Elicitation Tools

Book sharing is a common activity for young children, and wordless picture books are a well-known tool for eliciting naturalistic speech (Miller et al., 2006; Heilmann et al., 2015; Chaparro-Moreno et al., 2017). The legality of sharing copyright materials is a well-recognised challenge in medical testing (Feldman and Newman, 2013). Some of the best-known wordless picture books for speech elicitation (e.g., “Frog, where are you?”: Mayer, 2003) are protected by copyright. In the context of a lab-based, or home-visit study, a lab can purchase a small number of physical copies, which would then be re-used by all lab-visiting participants. However, in the transition to online studies, sharing a picture book by pdf or “broadcasting” the pages onscreen may infringe on the copyright of a published work, depending on the legal jurisdiction. To ensure our study materials would have applicability in a variety of geographical and technical contexts, we created an open access wordless picture book “Little Orangutan: What a Scary Storm!” (Styles, 2020b). As an added bonus, this open resource lowers usage barriers for researchers with limited funding.

After surveying a number of wordless picture books, the story was designed to focus on the emotions of an animal (an orangutan), in a familiar scenario (caught in the rain). The book is designed as a printable PDF, suitable for printing as an A4 folded booklet. For the online administration of the Talk Together Study, a screen-sharing version was created showing two-pages per spread. This picture book is an open access resource in our growing collection of open-source materials, the SESAME Research Tools [SESAME: Speech Elicitation for Spectral Analysis in Multilingual Environments, (Styles, 2021)].

### Video-Call Protocol

During task development, we tested several video call platforms for their stability, familiarity, and effectiveness. At the start of the study in June 2020, most parents in Singapore were familiar with the Zoom platform. The Data Collection team created a script and protocol for the recorded video call (Yogarrajah et al., 2021). A day before each scheduled video call, researchers would send a reminder text to the parent with the link to the password-protected Zoom meeting. In this reminder, parents were asked to pick a quiet spot for the call, and to join the video call using a large-screen device (e.g., tablets, laptops, iPads) so that the pictures would display at an appropriate size.

Unlike in completely asynchronous online studies (Rhodes et al., 2000), video-call studies allow a researcher to perform data quality checks before beginning the recording. First, the researcher confirmed that the shared screen was displaying correctly to the parent, on an appropriately-sized device. Second, the researcher checked that the audio quality was clear enough to enable later transcription. In some cases, parents switched devices, moved to a quieter part of their home, adjusted their home internet settings, or changed devices, to improve the audio fidelity of the parent and child’s voices relative to background noise.

**FIGURE 2:**
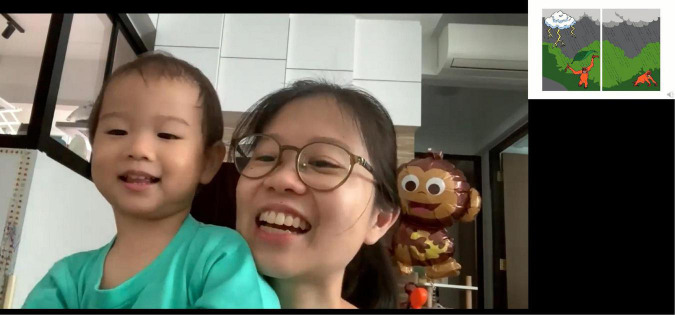
Illustration of the Videocall Storytime procedure for recording parent-child interactions. Left: zoom-captured image of a parent describing the onscreen wordless picture book. Right: onscreen images from the little orangutan book “What a Scary Storm!” image reproduced with permission (Yogarrajah et al., 2021).

After briefing, the researcher used the screensharing function to display the book, and switched off their camera to reduce distractions. Most parents chose to leave their camera on during the recording (see Figure 2). After obtaining verbal consent to record, the recording was started, and the Zoom platform displayed an onscreen notice to the parent. In place of book page-turning, parents were asked to say “next”, for the researcher to display the next slide in the deck, and a “jingling” sound was played.

At the end of the book sharing session, the researcher turned on their camera to conduct a debrief with the parents. Parents were asked whether they would consent to “audio release” in addition to their study consent. “Release forms” are common in creative industries so that a subject of a recording can agree to the specific ways in which their image or voice will be used in the future, and the decision is typically made after the subject knows what has been recorded (Crawford, 2009). In this case, participants were asked if they would allow for their audio recordings to be released to our research team’s open access repository known as the “Growing Collection of Human Voices” (Styles, 2020a). By granting permission for anonymised audio recordings to be released, parents allow uses beyond the original study (e.g., use in a public presentation, as a stimulus in a different study, or as a training dataset). Parents granted release for 385 out of 410 recordings we conducted (94%). Parents were reminded that they may choose to withdraw from the study at any time, or from the Growing Collection repository up until the anonymised recordings have been publicly archived.

Before ending the call, parents had an opportunity to ask questions, and would sometimes seek information about language development from our Data Collection team. These interactions are one of the intangible benefits of conducting online studies with live interactions.

After conducting the video call, the researcher completed an online log which triggered an automated email to the Intervention team to inform them that the parent was now ready to enter the next stage of the RCT. Recordings were downloaded to secured external hard drives and deleted from the Zoom cloud storage at the earliest possible time.

### Participant Remuneration Strategies

Participants were paid a token of appreciation upon completion of the video call at each time-point. We decided on cash payment rather than gift cards or vouchers, for the following ethical reasons: Firstly, many researchers who use vouchers are aware that not all participant tokens are redeemed during their eligibility period, meaning that the remuneration strategy may have hidden inequalities. Secondly, vouchers may be hard to utilise during a global pandemic, if the vendors in question are inaccessible, unable to make deliveries, or go out of business before voucher redemption. Finally, and most importantly, cash payments do not expire, and are maximally fungible. This means that remuneration is fair (all participants receive their remuneration), and equitable (remuneration can be used for anything including groceries, rent, or debt repayments). Cash payments therefore ensure equitable access to all participants, regardless of income level, and pandemic-related changes to income, housing, or financial status.

In Singapore, electronic payments are ubiquitous, including by registered accounts linked to mobile phone numbers (known as PayNow). The Data Collection team confirmed PayNow details with participating parents at the end of the video call, and filled in an online form while sharing the screen with the parents. The completion of this online form triggered an automated email to our lab’s manager who would then proceed with the wireless transfer. Participants were reimbursed upon completion of tasks at each time-point instead of a lump sum transfer at the end of the study.

Despite the ethical advantages of cash reimbursements, we have found that many parents in Singapore do not find cash payments to be strongly motivating. For this reason, in addition to cash reimbursement, we offered lucky draw tokens for each task they completed. We advertised 2 lucky draws, one for participants who completed all tasks at T1 and T2 (10 prizes, approximately 7% of all participants win a prize), and a second draw for participants who continued and completed T3 (5 prizes, 4% of all participants will win a prize). Each lucky draw comprised cash vouchers for the largest local supermarket chain, a children’s book, and a tote bag. While winning the lucky draws mean that the participation remuneration is unequally distributed, our blended model (cash for all, prizes for a few) ensured that all participants were paid an equitable amount prior to the motivational incentive of additional lucky draw rewards.

### Project Monitoring

A weekly all-hands project monitoring meeting was held to track progress. Each meeting opened with two check-in questions about unexpected events. Ethics check-in: “Were there any unexpected events that could have ethical consequences for the participants or their data?”; Procedural check-in: “Were there any unexpected events that could have consequences for the way we run our study, or what we can learn from it?”. During ethics check-ins, team members discussed situations like what to do when a parent became annoyed or scolded their child during the video call. In these cases, researchers paused the study and reassured parents that the video call was not a formal assessment of their parenting skills or their child’s general language abilities, but was a snapshot of language use in Singapore. Ethical training before beginning the study included the concept of *parental consent* coupled with the *child’s assent*. In line with best practice in studies involving minors (Royal College of Paediatrics and Child Health Ethics Advisory Committee, 2000), the Data Collection team reserved the right to stop the video call if they believed a parent was coercing a child to participate without assent.

## Deliverables and Progress

### Participant Retention

**FIGURE 3:**
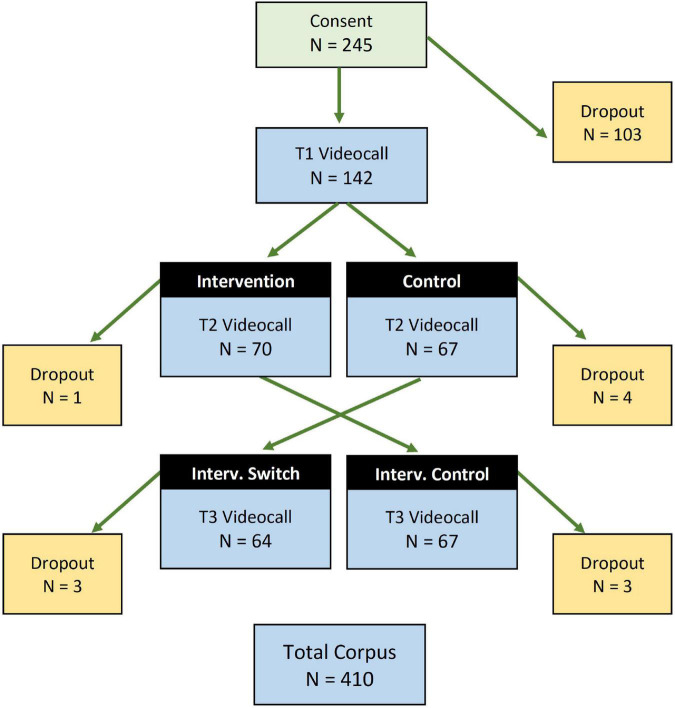
Flowchart of participants and dropouts at different stages in the study. Total corpus *N* = 410 recorded parent-child interaction videos.

A total of 245 participants with children aged between 8 and 40 months consented to participate in the remote study. Of these participants, 142 parent-child dyads completed baseline surveys and progressed to the video calls at T1 (see Figure 3), thereby beginning the micro-longitudinal study and RCT. Early withdrawals did not complete surveys in the chain asking screening questions (e.g., age of child) and detailed questions about the child’s language exposure, family language use, and parenting-related questions. Withdrawals likely indicated that a participant did not have time to participate fully.

Attrition in longitudinal research can be a substantial source of bias in medical and developmental studies (Reinwald et al., 2015; Pan and Zhan, 2020). Although internet-based studies may reduce the overhead required for a family to continue their participation in a longitudinal study, fully asynchronous internet research designs may actually increase dropout rates due to lower participant engagement. The use of video calls with researchers in our Talk Together study combines the ease of access of internet-based methods with the benefits of social interaction that are a part of face-to-face research designs.

Of the 142 parent-child dyads who progressed to the main micro-longitudinal study, we had extremely high participant retention to T2 (96%) and T3 (92% of total), demonstrating that live video call interactions, combined with close project monitoring, can mitigate against attrition in remote studies of this kind. With these low attrition rates across three time-points, we were able to record a total of 410 video calls, making this the largest collection of parent-child speech in Singapore. Transcriptions of the recorded video calls are underway and will be reported separately.

### Data Quality

Behavioural studies often have concerns about the fidelity of data collected online relative to lab-based studies, as participants could falsify eligibility criteria, “cheat” to improve their answers, or pay less attention when performing tasks unobserved (Woods et al., 2015; Rodd, 2021). Since our task involves a live video call with parent and child, researchers could monitor engagement, and check eligibility as they would in a lab-visit scenario. In addition, questions in the survey chain about language use in the home also acted as screeners and allowed us to detect ineligible participation (including one family who signed up the same child twice). Since the preregistered study measures will be derived from multilingual transcriptions of the parent-child interactions, our primary data quality concern was whether the audio track in the recording would be sufficiently clear to enable word-level transcriptions. When planning the original study, we conducted a pilot of the parent-child storybook protocol in a sound attenuating room, using Zoom H4n Pro precision audio recorders. Compared to this small sample of 11 parent-child dyads, the research team noted that the audio quality from the video calls was noisier and of more variable clarity, depending on hardware, internet connectivity, and position in the home. Technology failures like poor Wi-Fi connections, low battery and faulty devices sometimes disrupted data collection, leading to videocall rescheduling. However, since the protocol included an opportunity to change devices or locations, recordings did not proceed until they were judged to be of sufficient audio quality to be transcribed manually.

During periods when social distancing mandates were in place, most parents were working from home, and preschools and childcare services were sporadically unavailable. Unlike in lab-based studies, recordings were occasionally disrupted by other family members, which could disrupt the procedure. When this occurred, the researcher could offer to schedule the call for a later time, or continue the call with requests for the additional people to remain quiet. Data quality checks will continue as our in-lab transcribers monitor task adherence, and create a protocol for further exclusions if necessary.

### Reception of the Study by Parents

In general, at the time of the video call, parents gave positive feedback about participating in the study, indicating that the VideoCall Storytime protocol was suitable for parents. Many parents gave positive feedback about the wordless storybook “What a Scary Storm!”, stating that it was fun and interesting, and some even asked whether they could purchase a printed copy of the book.

In our preliminary analyses, almost all parents in the intervention condition reported that the tips provided during the intervention had changed their thinking about language use with their children, and/or their language behaviour with their infants and toddlers (c.f., Amran et al., 2021). Beyond these subjective impacts, transcriptions of the video calls are in progress to find out whether the intervention had a measurable impact on parent’s child-directed speech during the video call.

### Generalisability

Although the research context provided by Singapore’s multilingualism and high technological development is somewhat unusual in global contexts, the lessons learned during the design and delivery of the study have broad applicability for research teams interested in documenting or evaluating parent-child interactions, or other aspects development best captured through live interactions where a trained researcher is present. The procedures could be readily adapted for studies investigating toy play, theory of mind, numerical processing, spatial problem-solving, motor development, and a variety of other developmental milestones. We were able to conduct the study using Zoom, in part due to the prevalence of stable, high-bandwidth home internet connections in Singapore. Although these technological overheads may not be available in some research contexts, lower bandwidth versions of the protocol may be achieved by serving onscreen stimuli by a separate slide-sharing website and recording only audio during synchronous online sessions. One drawback of remote digital interactions may be recruitment bias toward those with higher SES and technological skills (Rodd, 2021). However, we believe these limitations are balanced by the ability for online studies to broaden participation among groups who may not otherwise be able to travel for an in-lab visit. In addition, our experience during this socially distanced COVID-19 pandemic has suggested this method is viable for reaching out to a variety of participants when physical travel is limited.

Lessons learned during the design, development, and running of the Talk Together Study are summarised in Table 1.

**TABLE 1:** Summary of lessons learned.

1	Recruitment strategies	Having an established virtual presence before recruitment (e.g., an active Facebook page) can help to build community trust in your studies.
		Online parenting groups are a great way to connect with potential participants, using posts and shares rather than paid ads.
		It is important to monitor social media for questions from potential participants before and during the consent procedure.
2	Consent and survey chain	A dedicated lab handset with mobile data, and lab-based social media accounts allow parents to ask questions and interact using asynchronous text-based messaging during consent, and throughout a study.
		Breaking up long surveys into smaller sections allows parents to break up the task into manageable chunks, with feedback on progress.
		Visual checklists help communicate study progress to participants.
		Automated emails to participants at each stage in the survey chain help them remember what they should do next.
		Automated emails to the research team allow tracking of task progress.
		Long surveys with a consistent format may be easier to complete using a clickable PDF, as this reduces “load time” in digital survey environments.
		Swapping between online surveys and PDF surveys that must be emailed back to the research team may cause some confusion for participants and needs to be monitored with care.
3	Video call scheduling	Reminder messages and calls help busy parents to remember stages in the study process they have missed or forgotten.
		Pre-call survey completion checks reduce data loss as no video call is conducted without the contextual data required for analysis.
4	Speech elicitation tools	Wordless picture books give a parent-child dyad something to focus on during their interaction. The stimulus is the same for all parents, but the choice of language(s), the level of complexity, and the choice of vocabulary is unconstrained.
		Open access research tools enhance the variety of contexts in which a tool can be used (including online), and lower barriers to use of the tool for researchers with limited funding.
		Wordless picture books reduce bias in a parent’s use of particular linguistic varieties, registers and speech styles. Study materials designed for use in multilingual or contact language contexts should be designed for that context, rather than imposing monolingual modes of language use as default.
5	Video call protocols	Synchronous video calls initiated by a researcher allow the research team to optimise the audio and video quality before beginning the recording. Video recordings provide helpful supplementary context for transcribers.
		Asking a participant to switch on a camera in their own home may be more complex in some contexts than others. For example, some religious communities may need to ensure non-participating members of the household are out of shot, or dressed differently for the duration of the call.
		Sensitivity to different home situations and flexibility should be built into the protocol, if possible (e.g., allowing a family to participate with their camera off if necessary), as it may facilitate broader participation.
		Using a single videoconferencing platform with all participants allows the research team to the protocol for that tool.
6	Participant remuneration	Cash (or wireless cash transfer) is the most ethical form of reimbursement as it is equitable and fungible.
		Lucky draws are inherently inequitable, but can be highly motivating.
		A combination of cash reimbursement and lucky draw ensures all participants are ethically reimbursed and provides additional motivation.
7	Project monitoring	All-hands check-ins provide an opportunity for ethical monitoring as well as project progress monitoring.
		All-hands check-ins allow all team members (including junior lab members) to share progress reports and contribute to project development throughout the research process.
		Building team feedback into the timeline of a study allows valuable opportunities to refine protocols during data collection and for future studies.
		Including opportunities for parents to give feedback on their impression of the study allows valuable opportunities to refine protocols while a study is still running, or to learn from participant experience.
8	Participant retention	Surveys for parents to complete in their own time have relatively high non-completion rates (a combination of ineligible respondents, and legitimate-but-busy participants). Using chained surveys with visual reminders sent to participants will increase rate of completion.
		Scheduling and conducting video calls are costly for a research team’s time and labour.
		Participants who were able to complete time-consuming surveys and a scheduled video call were highly likely to continue at later time-points.
		Studies that require both live interactions (e.g., video calls) and solo participation (e.g., survey completion) can use surveys as a screener before committing researcher time to live interactions.
9	Data quality	Combining the flexibility of online research with synchronous researcher-led methods allows research staff to optimise data quality before initiating the recording of a research data object
		Online recording conditions may not be as controlled as in-lab recording conditions, making them more suitable for some types of research measurements (e.g., transcription of speech, coding of behaviour) than others (e.g., precision eye-tracking, fine-grained acoustic analysis). Researchers should pilot their planned procedures to check that they meet the data quality required for their planned analysis before beginning a large-scale study.

## Conclusion

Over the 12 months of the Talk Together Study, our research team was able to make hundreds of recordings of naturalistic parent-child interactions using a novel procedure called Videocall Storytime. The procedure was contact-free, socially distanced, and possible to run during even the strictest lockdown conditions. Researcher-initiated video calls allowed for data quality checks to precede the onset of recording, and the interactions between parents and researchers provided supplementary motivation to continue in the longitudinal study. The lessons learned during adapting and running the study cover 10 domains of research design, monitoring and feedback: Recruitment strategies, Surveys and Questionnaires, Video call scheduling, Speech elicitation tools, Video call protocol, Participant remuneration, Project monitoring, Participant retention, Parental feedback, and Research team feedback. These lessons may have broad applicability in future research that extends the bounds of research with children beyond the constraints of face-to-face interactions between researchers, children, and their families.

## Data Availability Statement

Materials used in this study are publicly archived, and available here: Videocall Storytime Protocol (https://doi.org/10.21979/N9/0UYKJC) Language Experiences Overview (https://doi.org/10.21979/N9/XQUFEW) SHELLS: Supportive Home Environments for Language Learning – Survey (https://doi.org/10.21979/N9/RL5UMY) Talk Together – A 4 week programme of tips to enhance parent-child interactions (https://doi.org/10.21979/N9/W1D24L).

## Ethics Statement

The studies involving human participants were reviewed and approved by Nanyang Technological University IRB Board. Written informed consent to participate in this study was provided by the parent participants for themselves and their children.

## Author Contributions

FTW, SS, and SJS contributed to the design of the Talk Together Study and prepared the figures. ECY, SF, NSMS, and FTW contributed to the videocall storytime protocols. SS and FTW contributed to the project monitoring. FTW and SJS wrote the manuscript with contributions from all other authors. All authors approved the submitted version.

## Conflict of Interest

The authors declare that the research was conducted in the absence of any commercial or financial relationships that could be construed as a potential conflict of interest.

## Publisher’s Note

All claims expressed in this article are solely those of the authors and do not necessarily represent those of their affiliated organizations, or those of the publisher, the editors and the reviewers. Any product that may be evaluated in this article, or claim that may be made by its manufacturer, is not guaranteed or endorsed by the publisher.

## Footnotes

1
www.mturk.com

2
www.prolific.co
